# A systematic review, and meta-analyses, of the impact of health-related claims on dietary choices

**DOI:** 10.1186/s12966-017-0548-1

**Published:** 2017-07-11

**Authors:** Asha Kaur, Peter Scarborough, Mike Rayner

**Affiliations:** Centre on Population Approaches for Non-Communicable Disease Prevention, Nuffield Department of Population Health, University of Oxford, Oxford, England

**Keywords:** Health claims, Nutrition claims, Food labelling, Food choices

## Abstract

**Background:**

Health-related claims are statements regarding the nutritional content of a food (nutrition claims) and/or indicate that a relationship exists between a food and a health outcome (health claims). Their impact on food purchasing or consumption decisions is unclear. This systematic review measured the effect of health-related claims, on pre-packaged foods in retail settings, on adult purchasing decisions (real and perceived).

**Methods:**

In September 2016, we searched MEDLINE, EMBASE, PsychINFO, CAB abstracts, Business Source Complete, and Web of Science/Science Citation Index & Social Science Citation Index for articles in English published in peer-review journals. Studies were included if they were controlled experiments where the experimental group(s) included a health-related claim and the control group involved an identical product without a health-related claim. Included studies measured (at an individual or population level); actual or intended choice, purchases, and/or consumption. The primary outcome was product choices and purchases, the secondary outcome was food consumption and preference. Results were standardised through calculating odds ratios and 95% confidence intervals (CI) for the likelihood of choosing a product when a health-related claim was present. Results were combined in a random-effects meta-analysis.

**Results:**

Thirty-one papers were identified, 17 of which were included for meta-analyses. Most studies were conducted in Europe (*n* = 17) and the USA (*n* = 7). Identified studies were choice experiments that measured the likelihood of a product being chosen when a claim was present compared to when a claim was not present, (*n* = 16), 15 studies were experiments that measured either; intent-rating scale outcomes (*n* = 8), consumption (*n* = 6), a combination of the two (*n* = 1), or purchase data (*n* = 1). Overall, 20 studies found that claims increase purchasing and/or consumption, eight studies had mixed results, and two studies found consumption/purchasing reductions. The meta-analyses of 17 studies found that health-related claims increase consumption and/or purchasing *(OR 1.75, CI 1.60–1.91).*

**Conclusion:**

Health-related claims have a substantial effect on dietary choices. However, this finding is based on research mostly conducted in artificial settings. Findings from natural experiments have yielded smaller effects. Further research is needed to assess effects of claims in real-world settings.

**Trial registration:**

PROSPERO systematic review registration number: CRD42016044042.

**Electronic supplementary material:**

The online version of this article (doi:10.1186/s12966-017-0548-1) contains supplementary material, which is available to authorized users.

## Background

Poor diet is a leading cause of ill health. It has been estimated that 11.3 million deaths a year worldwide are attributable to dietary risk factors [1]. The World Health Organization recommends that adults consume at least five portions of fruit and vegetables a day, restrict their fat intake to 30% of the total energy intake, saturated fat to 10%, and free sugars to 5% of the total energy intake, and limit their salt intake to less than 5 g a day [2].

In order to address the burden of a poor diet food labels can be used to provide nutrition information to the consumer. People who read the nutrition information on food labels tend to have a healthier diet; however some consumers find the information difficult to understand and/or interpret [3]. Consumers would benefit from more interpretative aids to simplify the information provided on food labelling [3, 4].

Health and nutrition claims could potentially be used as interpretative aids. A health claim is ‘any claim which states, suggests or implies that a relationship exists between a food category, a food or one of its constituents and health’ [5]. Whereas a nutrition claim is ‘any claim that states, suggests or implies that a food has particular beneficial nutritional properties due to the energy, nutrients or other substances it contains, contains in reduced or increased proportions or does not contain’ [5]. It has been estimated that within Europe approximately 26% of pre-packaged foods carry a health or nutrition claim [6].

Health and nutrition claims (henceforth referred to as ‘health-related claims’) may help consumers identify healthier products if they are used responsibly [7, 8]. However, they also have considerable potential to mislead consumers [9, 10]. For example, consumers may attribute excessive health benefits to consuming a food with a claim (‘magic bullet’ effect) [11, 12]. They may incorrectly perceive a product carrying a health-related claim more positively than a product without a claim (positivity bias). Finally, they may incorrectly ascribe the product with positive attributes unrelated to the claim (‘heath halo’ effect) [13].

There is some contention on the effect of health-related claims on dietary choices. There is some evidence that health-related claims may increase consumption for example Wansink and Chandon (2006) [14] found that participants ate more of a snack food when it was described as ‘low fat’. However, other studies have found that health-related claims reduce consumptions as they lower consumers’ taste expectations [15, 16].

A variety of methods have been used to study the effect of health-related claims. Early research into the effects of health-related claims on dietary choices looked at the sales of products before and after a claim was introduced. For example, a number of studies examined population sales data of breakfast oats before and after a health claim was used and found that sales increased once a health-related claim was added to the packaging [17]. Whilst these types of natural experiments have substantial external validity the lack of control means that there may be other factors driving the sales increases for example promotional campaigns or price reductions.

In contrast to this, experimental studies in controlled environments allow for more precise manipulation of these factors and are easier to replicate compared to natural experiments. For example, discrete choice experiments in laboratory settings allow researchers to manipulate multiple attributes of a product and to then measure how these changes affect the participants’ choices. Product attributes are systematically manipulated and presented to the participants in choice sets. In conjoint analyses it is assumed that the participants make trade-offs for the attributes they value and through this the utility of each attribute can be estimated. However, to the best of our knowledge, these types of studies of the effect of health-related claims on dietary choices have not been reviewed systematically.

Previous systematic reviews on dietary choices have examined the role of nutrition labelling in dietary choices [3, 4]. These reviews found that nutrition labels can be used to guide choices although this varies by population subgroup. However, there have been very few systematic reviews that specifically examine the effect of health-related claims. Schemilt, Hendry, & Marteau (2017) [18] conducted a systematic review of the impact of nutrition claims on selection, consumption, and perceptions of food products but did not consider health claims in the review. Williams (2005) [12] conducted a systematic review on consumer understanding and use of health claims and found some evidence that claims may improve the quality of dietary choices. However, effects were not quantified.

Therefore, we conducted a systematic review of experimental studies to quantify the effect of health-related claims, on food labels in a retail setting, on adults’ dietary choices. Our primary outcome was the likelihood of choosing a product when a health-related claim was present compared to when such a claim was not present. Our secondary outcome was the percentage change (from when a health-related claim was present compared to when such a claim was not present) in measured, actual or intended, consumption and/or purchases.

## Methods

The protocol for this review was registered with PROSPERO in August 2016 (Systematic review registration number: CRD42016044042) [19].

### Identification of studies

The search strategy was created with input from an information specialist (NR) and designed to capture any study of the effects of food labelling. Terms related to participants or study designs were not included in the search strategy as we expected much heterogeneity. The searches were piloted in November 2015 and the finalised searches conducted in December 2015 and re-run in September 2016 to check for new studies. The search terms are presented in Appendix A. We searched MEDLINE, PsychINFO, Embase, CAB abstracts, Business Source Complete, and Web of Science/Science Citation Index & Social Science Citation Index. To be eligible for inclusion articles had to be written in English and published in a peer review journal. No date restrictions were placed on the search.

### Selection of studies

An article was included if it was a controlled experiment that examined the effect of health-related claims on food labels on adults’ actual food purchasing and/or consumption behaviour or intended behaviour. Pre- and post-studies that collected longitudinal individual level data or population level data on real shopping behaviour were eligible. The health-related claim had to be presented in a retail setting or scenario (e.g. supermarkets) and not a food service setting or scenario (e.g. menus, canteens etc.). For the purposes of this review an appropriate control was defined as the same product without a health-related claim but similar in all other aspects. Non-health related claims (e.g. taste or organic claims) were not considered to be appropriate control claims due to evidence of a taste/health association with food choices [15, 16].

The definitions and categorisations of health-related claims are those proposed by the International Network for Food and Obesity/non-communicable disease Research, Monitoring and Action Support (INFORMAS) [20] which are based on the definitions of the Codex Alimentarius Commission (Codex) [21]. Definitions and examples of sub-types of health-related claims are detailed in the Additional file 1. Only explicit health-related claims were considered in this review. Implicit claims, for example a picture of a person running or a heart shaped logo (without underlying nutritional criteria for its use), were not included. Health-related claims could be presented as text, a symbol or a combination of both.

Studies that solely examined children’s and/or adolescents’ dietary choices were not included, neither were studies that were concerned with the purchases of; infant and baby foods including follow-on milks, foods for specific nutritional uses, alcoholic beverages, and vitamins and mineral supplements. Studies that estimated the maximum monetary amount participants were willing to pay for a product with specific attributes were excluded. Studies that presented the health-related claim as part of a wider intervention (e.g. healthy eating initiatives, weight loss groups etc.) were also not included.

### Data extraction, synthesis, and analysis

The database search results were imported into Endnote V7. A single researcher (AK) completed the first screen of titles to remove any duplicate references and studies that were clearly unrelated to the systematic review. Full text articles were obtained when the title and abstract suggested that the study met the inclusion criteria. The full text was also sought when there was ambiguity about a paper’s relevance to the review. Another researcher (PS) assessed 10% of the references (minus records excluded at the title screen stage) in order to check for any disagreements in classification. Data was extracted into an Excel spreadsheet. A list of the column headings used can be seen in the Additional file 1. Where further information about a study was required the corresponding and/or the first author were contacted.

The Cochrane Risk of bias tool [22] was adapted and used to assess the study quality (Table 1). Studies were assessed for the following potential sources of bias; selection, performance, detection, recruitment, and funding.

**Table 1:** Risk of bias (quality) assessment: Cochrane risk assessment tool [22]

Bias domain	Source of bias	Health-related claims studies
Selection bias	a. Random sequence generation	Were participants/products randomised to the health-related claim condition?
	b. Allocation concealment	Were participants aware of claim allocation?
Performance bias	Blinding of participants and personnel	Were participants blinded to the aims of the study? (e.g. the impact of health-related claims on purchasing/consumption)
Detection bias	Blinding of outcome assessment	Were participants aware of the study outcomes?
Other bias	Anything else	How were participants recruited? Were participants/products representative of the target population? How was the study funded? Were there any conflicts of interest reported?

A two-step data analysis strategy was employed. First a sign test that indicated how the study addressed the primary research question ‘do health-related claims increase, actual or intended, consumption and/or purchasing?’ The second step was to quantify the effect by calculating an odds ratio for choice-based studies and/or percentage change for consumption and/or intent-rating scale (e.g. Likert scale ratings measuring purchase or consumption intent). Where possible, 95% confidence intervals were calculated (95% CI). Where studies reported a log-likelihood for choosing a product (sometimes referred to as ‘parameter estimates’) the results were exponentiated to calculate the Odds Ratio. Where results were presented, for the same population, for sub-types of health or nutrition claims we calculated a weighted average of the results. Parameter estimates for the entire population (i.e. not aggregated by participant characteristics) were used; where results were stratified a weighted average was calculated.

A random-effects meta-analysis was conducted due to the high level of heterogeneity between the studies. Data were analysed by claim type (health or nutrition claims) and by food category (based on UK Eatwell Guide categories [23]). Planned analyses by participant characteristics (e.g. gender and/or socioeconomic status) were not conducted as this data was not available for the studies included for meta-analyses. An influence analysis was conducted to assess if the omission of one study would greatly alter the results of the meta-analyses. Funnel plots were conducted to assess for publication bias. The results presented in the papers were standardised in Excel and the meta-analyses conducted in Stata v11 SE [24].

## Results

### Description of studies

#### Results of the search

**Fig. 1:**
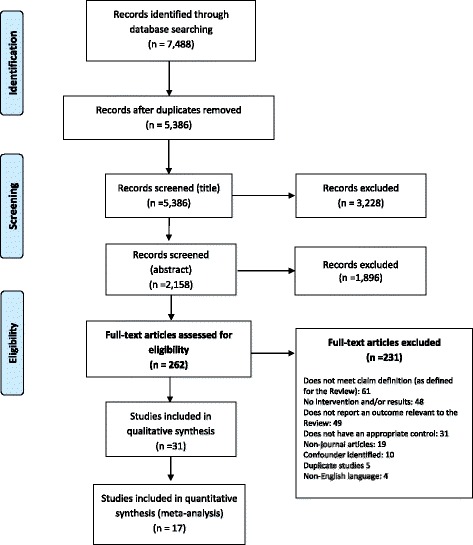
Prisma flow diagram

In total 5386 unique studies were identified through the database searches, of which 31 [14, 25–54] were deemed eligible for inclusion. The PRISMA flow diagram is provided in Fig. 1. The observed agreement between the two researchers on the 10% sample was 87.6% (170/194 decisions), kappa = 0.47 (95% CI 0.30–0.65). A kappa of 0.47 would be categorised as ‘moderate agreement’ [55]. Of the 24 studies where there was a disagreement, a single paper was in the final set of included studies (but not included for the meta-analyses).

Overall, 262 papers proceeded to the full paper review and their eligibility was assessed using the inclusion criteria outlined above. Following this, 231 papers were excluded. The most common reason for exclusion was that the study was not concerned with a health or nutrition claim as defined above (*n* = 61). A summary of the 31 [14, 25–54] included studies is provided in Table 2.

**Table 2:** Summary of included studies

First author (year)	Country	Study design and setting	Population	Analysis
Choice experiments				
Aschemann-Witzel (2010) [28]	Germany.	Repeated measures: non-hypothetical choice/purchase simulation. Conducted in a laboratory.	220 consumers.	Chi-squared test (proportion chosen carrying claim vs overall proportion not carrying claims).
Aschemann-Witzel (2013) [29]	Germany.	Repeated measures: realistic purchase simulation. Conducted in a laboratory.	210 consumers.	One-sample T-tests: (proportion chosen carrying claim vs overall proportion not carrying claims).
De Marchi (2016) [45]	USA.	Repeated measures: price (4 levels) x calories (3 levels) x health claim (with/without) x organic claim (with/without) x carbon trust logo (with/without). Online choice experiment.	173 primary food shoppers and consumers of yogurt.	Random parameter logit with an error component model.
De-Magistris (2016) [36]	Spain.	Repeated measures: price (4 levels) x nutrient claim (absent, reduced fat claim, low salt claim). Setting unclear, conducted in-person, participants seated individually.	217 primary food shoppers.	Random Parameters Logit (RPL) model.
Fernández-Polanco (2013) [37]	Spain.	Repeated measures: price (4 levels) x origin (2 levels) x harvest method (2 levels) x sustainability (2 levels) x health claim (2 levels) x safety (2 levels).	169 participants.	Heteroscedastic logit model.
Gracia (2009) [38]	Spain.	Repeated measures: price (2 levels) x brand (2 levels) x nutritional information panel (2 levels x claim (2 levels).	400 food shoppers.	Logit model.
Krystallis (2012) [42]	Greece.	Repeated measures: product type (2 levels) x claims (5 levels) x flavour (2 levels) x price (3 levels).	140 participants.	Heteroscedastic extreme value (HEV) model.
Van Wezemael (2014) [54]	Belgium, France, the Netherlands, and the UK.	Mixed design: between groups (nutrition or health & nutrition claim exposure), within group (claim, no claim) x price (4 levels). Conducted online.	2400 beef consumers, 600 participants from; the Netherlands, Belgium, France, and the UK.	Multinomial logit (MNL) model, error component (EC) logit model.
Ares (2010) [27]	Uruguay.	Repeated measures: type of yogurt (3 levels) x brand (3 levels) x price (3 levels) x claim (with/without).	104 yogurt consumers.	Multinomial logit model (MNL). MNL used to estimate part-worth utilities.
Barreiro-Hurle (2010) [30]	Spain.	Repeated measures: price (4 levels) x nutrition labelformats (2 levels) x claims (1 nutrient comparison, 1 disease reduction).	800 participants, consumers of sausages and yoghurt.	Random Parameters Logit (RPL) model.
Casini (2014) [33]	Italy.	Repeated measures: certification (4 levels) x site of production (4 levels) x health claim - (4 levels including no claim) x price (4 levels). Online survey.	260 Italian consumers.	Latent class choice model.
Contini (2015) [35]	Denmark and Italy.	Repeated measures: price (4 levels) x origin/site of production (4 levels) x health claim (8 levels −3 relevant to Review).	2024 participants, 51% Denmark, 49% Italy.	Latent class model. Cluster analysis: 8-class model.
Loose (2013) [44]	Australia.	Repeated measures: 8 attributes (levels ranging from 2 to 8): incl. Price (4 levels) and claims (3 levels). Conducted online.	1718 seafood consumers.	Scale adjusted latent class model.Aggregated multinominal logit model
McLean (2012) [47]	New Zealand.	Repeated measures: 4 factorial design: brand (3 levels) x FOP label (3 levels) x claim (3 levels) x sodium content (2 levels). Screen-based.	500 participants with hypertension, 191 participants without hypertension.	Multinominal logit regression model
Mohebalian (2012) [48]	USA.	Repeated measures: juice type (3 levels) x origin (3 levels) x health claim (2 levels) x price (continuous). Online survey.	508 participants.	Conditional logistic regression.
Mohebalian (2013) [49]	USA.	Repeated measures: fruit type x price x product origin, x health claim. Online survey.	1043 participants. Study 1: 535 participants. Study 2: 508 participants.	Conditional logit regression.
Experiments - purchase data				
Kiesel (2013) [39]	USA.	Five differentiated labelling treatments over a period of four weeks in each of five supermarkets, targeting microwave popcorn products.	Supermarket details: five treatment stores.	Summary statistics and difference-in-differences.
Experiments - measured consumption				
Roberto (2012) [52]	USA.	Randomised controlled experiment, between groups design (no label, Smart choices, a modified SC symbol with serving size). Conducted in a laboratory.	243 participants.	One-way ANOVA (continuous variables). Chi-squared tests (categorical outcomes).
Belei (2012) [31]	The Netherlands.	Randomised controlled experiment, between groups design, 3 conditions (incl. With/without claim).	109 undergraduate students.	ANOVA.
Carbonneau (2015) [32]	Canada.	Randomised controlled experiment, between groups design, 3 conditions (low fat, energy, no claim), take home meals.	160 women.	Mixed models for repeated measures used to compare impact of the experimental labelling groups on mean daily energy intake.
Koenigstorfer (2013) [40]	Germany.	Study 2: 1 factorial experiment (with claim/without) but without being made aware of perceived serving size and not observed by interviewer, conducted in a University.	Study 2: 135 students.	ANOVA.
Steenhuis (2010) [53]	The Netherlands.	Repeated measures: two conditions: with claim/without claim, 1 week washout period between. Conducted in a University.	31 female participants from the University community.	Paired sample t-tests.
Wansink (2006) [14]	USA.	Study 1: Between groups design (with claim/without), conducted during a University open day. Study 3: Between groups design (2 (regular versus low-fat label) × 3 (no serving label, “Contains 1 Serving” label, “Contains 2 Servings” label). Conducted in a cinema.	Study 1: 269 participants, students and their families visiting food science and human nutrition open day, aged 18 < .Study 3: 210 university staff, undergraduates, and graduate students.	ANCOVAs: consumption by label type (low fat versus regular).
Experiment (rating based)				
Ares (2008) [25]	Uruguay.	Repeated measures, factorial experimental design (4 × 4), resulting in a set of 16 food concepts.	104 participants.	ANOVA.
Ares (2009) [26]	Uruguay.	Repeated measures: three categorical factors: type of functional ingredient (2 levels) x name of the ingredient (2 levels) x claim (3 levels - No claim, ‘Enhanced function’ claim, ‘Reduced disease risk’ claim).	83 participants.	ANOVA.
Coleman (2014) [34]	UK.	Repeated measures, online survey.	122 volunteers.	ANOVA with a Bonferroni post-hoc test.
Kozup (2003) [41]	USA.	Between subjects design: 2 (heart-healthy, no claim) ×3 (nutrition information level with control). Mail survey.	147 participants, primary shoppers of household.	Multivariate and univariate
Lin (2015) [43]	Taiwan.	Between subjects design: randomly assigned to with or without claim.	300 students and office workers	ANOVA.
Maubach (2014) [46]	New Zealand.	Repeated measures: 4 FOP summary indicators, ×3 nutrition profile levels, × 3 product claim levels (no claim, nutrient-content, health claim), ×4 flavours. Conducted online.	768 participants.	Odds ratio.
Moon (2011) [50]	USA.	Between subjects design, randomly assigned to treatment: (1) FDA permitted health claims (2) same claim without FDA approval (3) no information. Online survey.	3456 participants.	Logistic regression, t-test.
Orquin (2015) [51]	Denmark.	Between subjects design, realistic product photographs shown 1 at a time.	STUDY 3: 204 participants, recruited online.	Linear regression.

#### Types of studies

European studies were the most common studies with four studies from Spain [30, 36–38], three from Germany [28, 29, 40], two from the Netherlands [31, 53], and single studies from Denmark [51], Greece [42], Italy [33], and the UK [34]. There were two studies that used multiple countries; Contini et al. (2015) [35] compared consumer behaviour of participants in Denmark and Italy, and Van Wezemael et al. (2014) [54] investigated consumer preferences in Belgium, France, the Netherlands, and the UK. There were eight studies [14, 32, 39, 41, 45, 48–50, 52] conducted in North America, one of which was conducted in Canada [32]. Three studies were conducted in Urguay, South America [25–27], one study was conducted in Taiwan [43], Australia [44], and two in New Zealand [46, 47].

The most common study type was choice experiments (*n* = 15 [27–30, 33, 35–38, 42, 44, 45, 47–49, 54]), of these ten studies included conjoint analyses that were relevant to the review [27, 30, 33, 35, 36, 44, 45, 47–49]. There were nine experiments that involved participants rating, on a Likert scale, their intention to purchase or consume products [25, 26, 34, 41, 43, 46, 50–52], and six experiments that involved measuring how much participants consumed under different claim conditions [14, 31, 32, 40, 52, 53]. A single study used sales data to measure the effect of health-related claims [39].

#### Types of products and claims

There were eight studies that examined nutrition claims [14, 32, 36–39, 42, 47], 12 studies that examined health claims [27, 35, 40, 41, 43–45, 48–50, 52, 53], and 11 studies examined both health and nutrition claims [25, 26, 28–31, 33, 34, 46, 51, 54]. There was one study that measured the effects of health-related claims on ‘Fruits and Vegetables’ [48] whereas there were nine studies that examined ‘Foods High in Fat and/or Sugar’ [26, 31, 33, 35, 38–40, 42, 53], five studies examined ‘Beans, Pulses, Fish, Eggs, Meat and other Proteins’ [37, 44, 47, 50, 54], three studies on ‘Potatoes, Bread, Rice, Pasta and Other Starchy Carbohydrates’ [34, 46, 52], four studies on ‘Dairy and Alternatives’ [27, 36, 45, 51], and two studied ready meals [32, 41]. Six studies looked at multiple categories of foods [14, 25, 28–30, 49].

#### Outcomes

##### Likelihood of selecting product with health-related claim

**Fig. 2:**
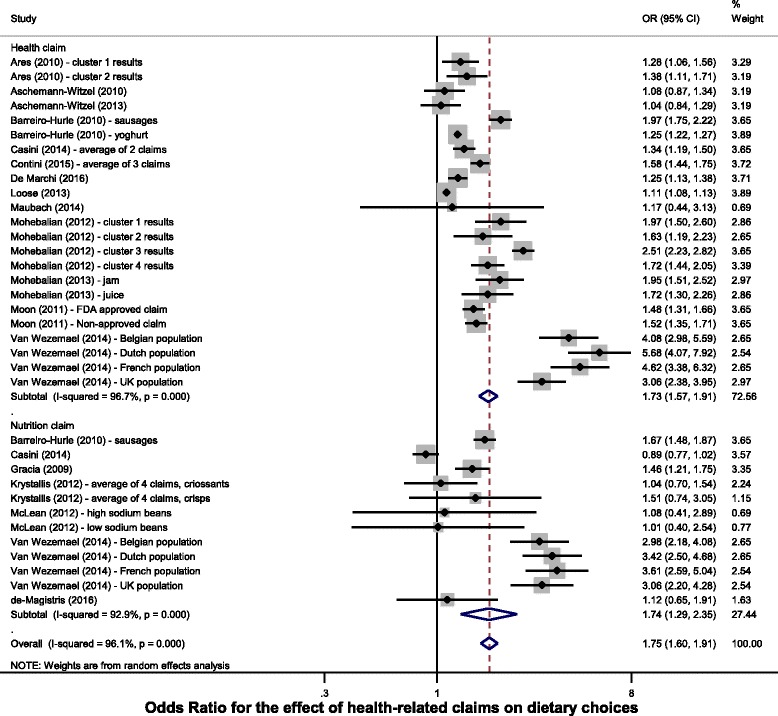
Forest plot for the effect of health-related claims on dietary choices, by claim type

In total, 16 studies [27, 28, 30, 33, 35–38, 42, 44–49, 54] reported the likelihood of choosing a product when a health-related claim was present, one study presented the percentage chosen of products with a health-related claim [29]. These results have been transformed into odds ratio where the comparator was always the same product without any claims (Table 3). Meta-analyses on the 17 studies (Fig. 2) found that products carrying health-related claims were more likely to be purchased or consumed than an identical product without a claim (OR 1.75, 95% CI 1.60–1.91). The effect was similar for nutrition claims (OR 1.74, 95% CI 1.29–2.35) and health claims (OR 1.73, 95% CI 1.57–1.91).

**Table 3:** Likelihood of selecting a product with a health-related claim

First author (year)	Outcome measure	Comment	Forced choice?	Product category	Claim sub-type (nutrient/target - health relationship)	Results: OR (95% confidence intervals)	Support the hypothesis?
Ares (2010) [27]	Part-worth utilities: multinomial logit regression.	Cluster analysis. Cluster 1 more diet and health concerned than cluster 2.	Yes	Yogurt.	RDR (fibre - cancer), (antioxidants - heart disease + cancer).	C1: 1.28 (1.06, 1.56)	Y
						C2: 1.38 (1.11, 1.71)	
Aschemann-Witzel [28](2010)	Proportion of products that carry claims & chosen.	OR calculated from the number of choices of a product with a claim and the number of expected choices of products with a claim, if the claim was chosen at random.	No	Yogurt, muesli, pasta.	HCs: NOF (calcium + vitamin D - bones/teeth), (folic acid - mental function), (fibre - bowel function), RDR (calcium + vitamin D - osteoporosis), (folic acid - dementia), (fibre - cancer).	1.21 (0.98, 1.43)	Y
Aschemann-Witzel (2013) [29]	Percentage products with claim chosen (number of choices) .	As Aschemann-Witzel (2010).	No	Yogurt, breakfast cereal, pasta.	NCs: Ncon (calcium, vitamin D), NOF (calcium, vitamin D - osteoporosis, Ncon (folic acid), NOF (folic acid - brain/mental functions).	1.10 (0.87, 1.32)	Y
Barreiro-Hurle (2010) [30]	Coefficient: random parameter logit.	Same population tested both products.	No	Pork Frankfurter sausage.	Ncon (fat)	1.67 (1.48, 1.87)	Y
					RDR (CVD)	1.97 (1.74, 2.24)	
					Ncon (fat) & RDR (CVD)	0.58 (0.49, 0.69)	
				Yogurt.	RDR (CVD)	1.25 (1.21, 1.28)	
Casini (2014) [33]	Parameter estimates: conditional logit model.	Average of NOF and RDR used for meta-analysis as same products and population.	No	Olive oil.	NOF (polyphenols - oxidative stress)	1.44, (1.29, 1.60)*	Y + N
					RDR (polyphenols - cholesterol)	1.23 (1.09, 1.39)*	
					*Average*	*1.33 (1.19, 1.49)*	
					HRIC (polyphenol)	0.88 (0.77, 1.01)	
Contini (2015) [35]	Parameter estimates: latent class model.	Average used as same product, population, and claim-sub type	No	Olive oil.	RDR (polyphenols - blood lipids)	1.41 (1.27, 1.57)*	Y
					RDR (olive oil - CHD)	1.66 (1.54, 1.80)*	
					RDR (olive oil - blood lipids)	1.70 (1.52, 1.89)*	
					*Average*	*1.58 (1.44, 1.75)*	
De Marchi (2016) [45]	Parameter estimates: random parameter logit with an error component.	Results adjusted for time preferences. Interaction terms not included.	No	Yogurt.	RDR (saturated fat & cholesterol - heart disease)	1.25 (1.13, 1.38)	Y
de-Magistris (2016) [36]	Parameter estimates: Random Parameters Logit model.	.	No.	Cheese.	Ncom (fat)	2.23 (0.01, 570.66)	
					Ncom (sodium)	0.56 (39.38, 0.01)	
Fernández-Polanco (2013) [37]	Coefficients: conditional logit model, (+WTP).	Was not included in meta-analyses as SE not reported.	No	Fish (seabream).	Ncon (omega-3)	1.63	Y
Gracia (2009) [38]	Coefficient: Parameters Logit model.	Interaction terms not included.	No	Breakfast cookies.	Ncon	1.46 (1.21, 1.75)	Y
Krystallis (2012) [42]	Coefficient (+WTP).	Averaged by product as same population and same claim sub-types.	No	Crisps.	Ncon (calcium)	2.31 (1.07, 5.00)*	Y + N
					Ncon (vitamins)	1.86 (0.92, 3.75)*	
					Ncon (omega-3 fatty acids)	0.77 (0.39, 1.49)*	
					Ncon (fibres)	1.54 (0.75, 3.18)*	
					*Average*	*1.50 (0.73, 3.07)*	
				Croissant.	Ncon (calcium)	1.31 (0.85, 2.00)*	
					Ncon (vitamins)	1.44 (0.99, 2.09)*	
					Ncon (omega-3 fatty acids)	0.74 (0.51, 1.07)*	
					Ncon (fibres)	0.83 (0.56, 1.22)*	
					*Average*	*1.04 (0.70, 1.53)*	
Loose (2013) [44]	Aggregated multinomial logit model/part worth utility estimates		No	Oysters.	Logo (Heart tick)	1.11 (1.08, 1.13)	Y
Maubach (2014) [46]	Hazard ratio: binominial logit regression.	OR for health claim scaled by OR for ‘no claim’. Results for NC not included. Interactions not included	Yes	Breakfast cereal.	RDR (wholegrain - cholesterol)	1.17 (1.13, 1.22)	Y
McLean (2012) [47]	Utility: multinomial logit regression (no FoP label model used)	600 participants 300 with hypertension, 300 without. Recruited from same database Averaged by product as same population and same claim sub type.	Yes	Low sodium Baked beans.	Ncom (sodium)	0.66 (0.53, 0.83)*	Y + N
					Ncon (sodium)	1.55 (1.22, 1.97)*	
					*Average*	*1.11 (0.88, 1.45)*	
				High sodium baked beans.	Ncom (sodium)	1.21 (0.94, 1.55)	
Mohebalian (2012) [48]	Odds ratio	Cluster analysis	No	Fruit juice.	NOF (antioxidants - immune system).	C1: 1.98 (1.51, 2.59)	Y
						C2: 1.63 (1.18, 2.24)	
						C3: 2.50 (2.2.4, 2.79)	
						C4: 1.72 (1.44, 2.06)	
Mohebalian (2013) [49]	Coefficient	Different populations for each product. Results adjusted for demographic characteristics.	Yes	Elderberry jelly.	NOF (antioxidants - immune system).	1.96 (1.52, 2.52)	Y
				Elderberry juice.	NOF (antioxidants - immune system).	1.71 (1.31, 2.25)	
Moon (2011) [50]	Rating: willingness to try 5 point scale	Different populations for each claim.	No	Soy foods.	RDR (protein - heart disease) FDA approved.	1.48 (1.32, 1.66)	Y
					RDR (protein - heart disease) Not FDA approved.	1.52 (1.35, 1.71)	
Van Wezemael (2014) [54]	Parameter estimates: error component model (best fit) (+multinomial logit model, +WTP)	Average by claim sub-type, same product, results by country.	No	Lean beef steak.	Ncon (iron), Ncon (fat), Ncon (protein).	NL: 3.42 (2.50, 4.69)	Y
						BE: 2.98 (2.17, 4.10)	
						FR: 3.61 (2.61, 5.00)	
						UK: 3.06 (2.18, 4.30)	
					NOF (iron - cognitive function), NOF (saturated fat - cholesterol), NOF (protein - muscle).	NL: 5.68 (4.06, 7.95)	
						BE: 4.08 (3.00, 5.56)	
						FR: 4.60 (3.38, 6.26)	
						UK: 3.06 (2.37, 3.97)	

*Abbreviations*: *HC* Health claim, *NC* Nutrient claim, *HRIC* health-related ingredient claim, *Ncon* nutrient content, *Ncom* nutrient comparative claim

*NOF* nutrient and other function, *RDR* reduction disease risk, *CVD* cardiovascular disease, *CHD* coronary heart disease, *FDA* USA Food and Drug Administration, *C#* cluster/class

Forced choice: No = participants were able to select neither products/no buy option

Where multiple OR are given, the *OR was NOT used in the meta-analyses

**Fig. 3:**
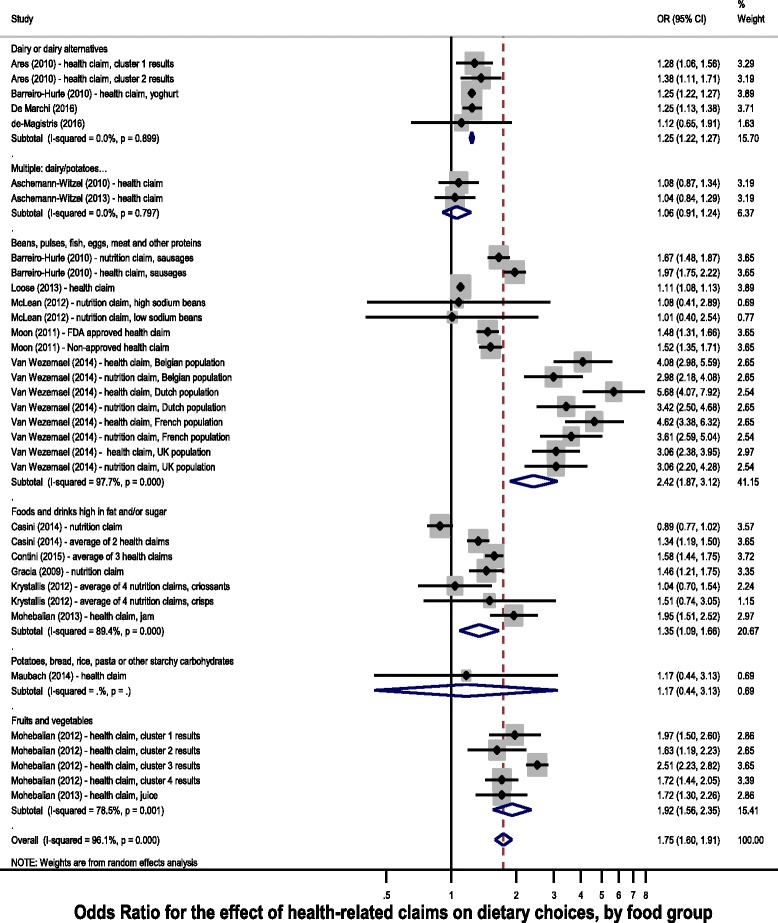
Forest plot for the effect of health-related claims on dietary choices, by eatwell food group

Analyses by food category (Fig. 3) found large effects for claims on products categorised as ‘Beans, Pulses, Fish, Eggs, Meat and other Proteins’ (OR 2.42, 95% CI 1.87–3.12), and ‘Fruits and Vegetables’ (OR 1.92, 95% CI 1.56–2.35), moderate effects for ‘Foods High in Fat and/or Sugar’ (OR 1.35, 95% CI 1.09–1.60), ‘Dairy and Alternatives’ (OR 1.25, 95% CI 1.22–1.27), and ‘Potatoes, Bread, Rice, Pasta and Other Starchy Carbohydrates’ (OR 1.17, 95% CI 0.44–3.13), and smaller, non-significant effects for multiple categories (‘Dairy and Alternatives’ & ‘Potatoes, Bread, Rice, Pasta and Other Starchy Carbohydrates’, OR 1.06, 95% CI 0.91, 1.24).

Figure 2 notes: Results have been aggregated when the same claim type (Health/Nutrition) has been used on the same product, and on the same population.

Studies appear multiple times if results for different populations have been presented. For example, Van Wezemael (2014) presented results for health claims and, with a different population, health and nutrition claims combined, for 5 countries.

Where studies present results for the same population but multiple claim sub-types an average has been calculated. For example, Casini (2014) presented the effect of two health claims and one nutrition claim on one population. An average of the health claim was calculated and a separate value for the nutrition claim was also included.

##### Change in preference or consumption of a product when a health-related claim was present

Products carrying health-related claims increased actual or intended purchasing/consumption by 8.9% (95% CI −4.9%, 22.6%, 10 studies) (Table 4). Health claims lead to a 9.8% increase (95% CI −8.4, 30.0), and nutrition claims lead to a 7.8% increase (95% CI −15.2, 30.8). The averages were then stratified by the outcome measure used. Studies that reported a rating scale outcome, such as the Coleman et al. study [34] which used a 5 point rating scale of purchase intent where 1 equalled “definitely” would not buy’ and 5 equalled ‘definitely would buy’, reported, on average, a 12.6% increase (95% CI 6.1%, 19.0%), whereas studies measuring consumption reported a 5.6% increase (95% CI −13.6%, 24.8% - five studies). A single study reporting store-level sales reported a 16.1% increase (95% CI 12.0, 20.2%).

**Table 4:** Change in preference or consumption of a product when a health-related claim was present

First author (year)	Outcome measure	Product category	Claim sub-type (nutrient/target - health relationship)	Results	Does it support the hypothesis?
Ares (2008) [25]	Rating: willingness to try, 7-point Likert scale	Yogurt	NOF (antioxidants)	−3.77% (−5.91%, −1.63%)	Y + N
			Ncon (fibre)	-3.34% (−5.48%, −1.20%)	
			Ncon (fat)	-6.80% (−8.94%), (−4.66%)	
		Milk desserts	NOF (antioxidants)	-2.98% (−5.20%, −0.75%)	
			Ncon (fibre)	-2.77% (−5.00%, −0.55%)	
			Ncon (fat)	-1.81% (−4.04%, 0.41%)	
		Bread	NOF (antioxidants)	-4.03% (−6.32%, −1.74%)	
			Ncon (fibre)	-0.70% (−2.99%, 1.59%)	
			Ncon (fat)	-4.21% (−6.50%, −1.92%)	
		Mayonnaise	NOF (antioxidants)	-12.12% (−14.63, −9.60)	
			Ncon (fibre)	-19.86% (−22.37, −17.34)	
			Ncon (fat)	−1.01% (−3.52%, 1.51%)	
Ares (2009) [26]	Rating: willingness to try, 7-point Likert scale	Milk dessert	NOF (fibre - calcium absorption + beneficial gut bacteria),(antioxidant - fat oxidation + cellular health)	+29.37%	HC: Y NC: N
			RDR: (fibre - cancer), (antioxidants - heart disease + cancer)	+31.47%	
			Ncon (fibre, b-glucans)	+16.29%	
			Ncon (antioxidant, flavoids)	+14.06%	
Belei (2012) [31]	Mean amount consumed	Chocolate	Ncon (fat)	+38.4% (25.0%, 51.7%)	Y + N
			NOF (cacao - antioxidant)	-34.3% (−41.3%, −27.3%)	
			Replication study:		
			Ncon (fat)	+ 43.4% (18.5%, 68.2%)	
			NOF (cacao - antioxidant)	-47.2% (−54.4%, −39.9%)	
			Ncon (low cholesterol	−49.5% (−54.6%, −44.4%)	
Carbonneau (2015) [32]	10 day mean energy (kcal) intake	.	Ncon (fat)	+3.4% (−2.1%, 8.9%)	Y
			Ncon (energy)	+ 3.9% (−1.9, 9.8%)	
Coleman (2014) [34]	Rating: purchase intent, 5-point Likert scale	White bread.	HRIC or GHC/Prebiotic	+17.6% (11.2%, 24.0%)	Y
			NOF (satiety)	+ 1.2% (−5.7%, 8.1%)	
			NOF (weight)	+ 14.9% (6.4%, 23.4%)	
			RDR (cancer)	+ 13.3% (5.3%, 21.4%)	
			NOF (minerals)	+ 22.0% (15.1%, 28.9%)	
Kiesel (2013) [39]	Sales over 4 weeks for pre-exposure and exposure period.	Microwave popcorn.	NUTRIENT CLAIMS	+16.1% (12.0%, 20.2%)	Y
			Ncon (energy)	+ 25.4%	
			Ncon (fat)	14.6% + 3.2%	
			Ncon (fat –FDA)		
Koenigstorfer (2013) [40]	Mean amount consumed	Savoury snack (trail mix)	GHC (Fitness)	+149% (110.9%, 186.2%)	Y
	N serving themselves:			OR 4.4 (3.6, 5.1)	
Kozup (2003) [41]	Rating: purchase intent, 7-point Likert scale	Frozen ready meal (lasagne)	RDR (saturated fat + cholesterol - CHD), Logo (Heart healthy - novel logo with description provided)	15%	Y
Lin (2015) [43]	Purchase intent rating	Tea drink	NOF (weight loss)	+10.22% (−20.9%, 41.4%)	Y
Roberto (2012) [52]	Rating: purchase intent, 9-point Likert scale	Breakfast cereal	Logo: Servings per pack	+16.3% (−2.7%, 35.4%)	Y + N
			Logo: Serving size	+ 23.7% (3.8%, 43.6%)	
			Buying for children		
			Logo: Servings per pack	+20.4% (0.8%, 40.1%)	
			Logo: Serving size	+ 16.6% (−2.3%, 35.4%)	
			Logo: Servings per pack	-0.3% (−13.9%, 13.3%)	
	Meant amount consumed: Total cereal + milk eaten (grams) Cereal poured (grams)		Logo: Serving size	+ 5.8% (−9.4%, 21.0%)	
Steenhuis (2010) [53]	Mean amount consumed	Chocolate mousse cake	Logo (Choices)	−7.4% (−21.7%, 6.9%)	N
Wansink (2006) [14]	Study 1: mean calories served	Chocolate and granola.	Ncon (fat)	Study 1: +28.4%	Y
	Study 3: mean calories consumed			Study 3: +50.1%	

The percentage change in preference/consumption differed by food groups; on average, health-related claims on ‘Dairy and Alternatives’ products led to a 5% reduction, whereas a 10% increase was observed for ‘Potatoes, Bread, Rice, Pasta or Other Starchy Carbohydrates’, a 12 point increase was observed for ‘Foods High in Fat and/or Sugar’, and a 7% increase for ‘Composite Foods’.

Seven studies reported purchase/consumption intent-rating scale outcomes where a higher rating indicated a greater intention to purchase and/or consume the product [25, 26, 34, 41, 50–52], however all used different scales i.e.; 7-point [25, 26] or 5-point [50] willing to try scales or, 5-point [34], 7-point [41, 51], or 9-point purchase intent scales [52]. Five of these studies reported increases in intent when a health-related claim was present [26, 34, 41, 50, 52] ranging from +1% [34] to +52% [50]. Coleman [34] tested five types of health claims on white bread and found that some claim types had a stronger effect than others. For example a nutrient and other function claim related to satiety led to a 1% intent increase (non-significant) whereas a similar nutrient and other function claim related to mineral content led to a 22% intent increase (95% CI 15%, 29%). Ares [25] found a reduction in intent when health and nutrition claims were presented on yogurts, desserts, bread and mayonnaise.

Five studies [14, 31, 32, 40, 53] reported the mean amount of food consumed in different health-related claim scenarios. Belei, Geyskens, Goukens, Ramanathan, & Lemmink (2012) [31], Koenigstorfer, Groeppel-Klein, Kettenbaum, & Klicker (2013) [40], Steenhuis, Kroeze, Vyth, Valk, Verbauwen, & Seidell (2010) [53], and Wansink & Chandon (2006) [14] all measured the mean amount (in weight) of food consumed, whereas Carbonneau et al. (2015) [32] measured the mean nutrient intake over a 10 days period. Despite reporting similar outcome measures there was still considerable variance in the in the average food consumption in the five studies. For example, when nutrition claims were present there was a 3–4% increase in consumption of ready meals [32] and a 28–50% increase in chocolate consumption [14], but a 149% increase in consumption of trail mix when a health claim was present [40]. Steenhuis et al. (2010) [53] examined the effect of the Choices health logo [56] on a chocolate dessert and found a 7% reduction (not statistically significant) in consumption. Belei et al. (2012) [31] also studied the effects of health-related claims on a chocolate product and found that a 38% increase in consumption when a nutrition claim was present and a 34% reduction when a health claim was present. Belei et al. then replicated this study and had similar results for the effect of a nutrition claim (43% increase) and found a larger reduction when a health claim related to antioxidants was present (−47%), and an even larger reduction with a low cholesterol claim (−50%).

In two studies Aschemann-Witzel et al. [28, 29] reported the proportion of products with a health-related claim that were chosen from a selection of products and found a 2–5% increase in the number of choices of products than if products were chosen at random.

### Risk of bias

The risk of bias table is available in the Additional file 1. We identified 13 studies as being at risk of selection bias due to the use of research panels for recruitment [34, 36, 41, 42, 44–51, 54], and for four studies the method of recruitment was not clear [28, 31, 35, 38]. For studies involving participants, most used random allocation and/or random sequence generation. The majority of the studies were at risk of performance bias as just three studies [14, 32, 51] used cover stories to reduce demand characteristics. For example, Wansink & Chandon (2006) [14] compared the amount of granola consumed when it was labelled as ‘low fat’ to when it was labelled as ‘regular’ but informed participants that the purpose of the study was to rate a video to reduce the likelihood that participants would alter their behaviour in accordance to the study aims.

The target population was often not stated in the paper; however 10 studies [27, 29, 30, 35, 37, 38, 48–50, 54] found that their participants’ characteristics fit well with national census data. No studies explicitly listed any conflicts of interest due to industry funding.

**Fig. 4:**
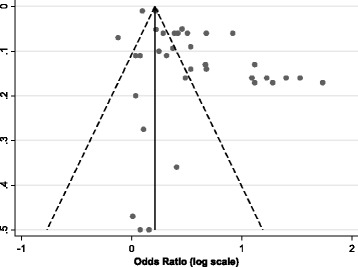
Funnel plot for publication bias (with pseudo 95% confidence limits)

Tests revealed a high level of heterogeneity in the results (I-squared: overall 96%, health claims 97%, nutrition claims - 93%). A funnel plot showed strong asymmetry suggesting that there was publication bias (Fig. 4).

An influence analysis was conducted to assess if the omission of one study would greatly alter the results of the meta-analyses. Overall, four studies had a large effect on the results, the omission of which affected the estimated effect size by more than 5%. When the Van Wezemael et al. study [54] was omitted it led to an 18% reduction to the overall estimated effect size (OR 1.43, 132–1.55), the omission had a greater impact on the effect size for nutrition claims (30% reduction, OR 1.22, 0.93–1.60) than for health claims (18% reduction, OR 1.49, 1.36–1.62). Three other studies also led to greater than 5% change in the estimated effect size for nutrition claims - omitting Casini et al. (2014) [33] led to a 10% increase (OR 1.91, 1.47, 2.48), whereas omitting Krystallis & Chrysochou (2012) [42] and Mclean, Hoek, & Hedderley (2012) [47] led to smaller increases (OR 1.85, 1.33–2.59, and OR 1.84, 1.34–2.54, respectively).

## Discussion

### Summary of main results

Results of choice experiments (without actual purchasing of foods) suggest that products carrying a health-related claim are 75% more likely to be chosen than an identical product without a health-related claim (OR 1.75, 95% CI 1.60–1.91). This effect is similar for nutrition claims (OR 1.74, 95% CI 1.29–2.35) and health claims (OR 1.73, 95% CI 1.57–1.91). The effect varies by the category of the food that the claim was presented on: larger effects were seen for health-related claims on products categorised as ‘Beans, Pulses, Fish, Eggs, Meat and other Proteins’ (OR 2.42, 95% CI 1.87–3.12) or ‘Fruits and Vegetables’ (OR 1.92, 95% CI 1.56–2.35), than for ‘Foods High in Fat and/or Sugar’ (OR 1.35, 95% CI 1.09–1.60) or other food categories.

The results should be viewed with caution due to the risk of bias associated with the studies, the high degree of heterogeneity in study findings and the potential risk of publication bias revealed by the funnel plot. Overall, the results that have been derived from studies using continuous outcomes (ratings, sales, amount consumed etc.) demonstrate much more conservative results than those that have been estimated by conjoint analyses. Averages of such studies estimated that health-related claims led to just an 8.9% (95% CI −4.9%, 22.6%) increase in purchases/consumptions. Kiesel & Villas-Boas (2013) [39] examined the effect of nutrition claims (on shelf labels) on real-life purchases of popcorn products by examining, across five stores, the difference in sales in between when a shelf-label intervention was present and was it was not. They found that low calorie claims increased sales but low fat labels decreased sales. When these results were standardised for this systematic review we estimated the overall effect of nutrition claims to increase sales by 16.1% (95% CI 12.0, 20.2), much lower than 75% increase estimated from the meta-analyses.

The results from the meta-analyses suggest that health and nutrition claims have a similar effect on dietary choices. This would be supported by previous research on health-related claims which suggests that consumers often do not clearly distinguish between health and nutrition claims [12].

The studies included in this systematic review cover a range of foods and all of the food groups (as categorised by the UK Eatwell Guide) were represented, however there was only one study [48] that examined the effect of health-related claims on fruits and vegetables. Mohelbalian, Cernusca, & Aguilar (2012) conducted a choice experiment examining health claims on a fruit juice product and found that the odds of choosing the product with a health claim varied by how health conscious the consumer was and whether they already consumed the product. Less health-conscious consumers who already consumed the fruit juice were more likely to choose the product with the health claim (OR 1.63, 95% CI 1.19,2.23) but health-conscious consumers who did not already consume the product had a much higher odds of choosing the product when a health claim was present (OR 2.51, 95% CI 2.23, 2.82). This suggests that consumer attributes, such as lifestyle traits, may be an important mediator of the effect of health-related claims.

Although each data line in the meta-analysis is drawn from either a separate experiment or a separate population (or both) many were conducted with similar methods and hence potentially similar biases (e.g. Van Wezemael, 2014 [54]). In the random effects models that we used in this paper we did not adjust for potential correlation between estimates produced with similar methods. In a multilevel meta-analysis (35 results nested in 17 studies) of the combined effect of health and nutrition claims the effect size reduced from 1.75 to 1.41 (95% CI 1.20, 1.67). Such a method accounts for study-level correlation [57], however in this case may over-adjust since the data lines are all drawn from either separate experiments or separate populations or both.

Whilst choice experiments are able to isolate the effect of the claim from other competing influences (e.g. price, brand, store factors etc.), they are conducted in an artificial context and therefore may have limited external validity. Similarly, in these choice experiments participants are asked to choose between the product with a claim and the control product (without a claim). It is unclear whether these choices would equally translate into real-world purchases made with the participants’ own money, particularly when other factors such as positioning, package design, and brand factors are likely to play a role.

### Limitations of the review

This systematic review is the first, that we are aware of, that has attempted to quantify the effect of health-related claims on dietary choices using odds ratios and/or estimating the percentage change in consumption, willingness to purchase/consume, or actual sales. We have used an established taxonomy for the classification of claims which is compatible with EU and international regulations.

As there has been a large amount of research published on various aspects of health-related claims (e.g. claim understanding, substantiation, recognition etc.), during the abstract screening stage studies were only included if the abstract mentioned one of the following outcomes; choices, purchases, or consumption. It is possible that studies that did not mention an outcome relevant to the systematic review went on to present relevant results in the full paper – such studies would not have been included.

Furthermore, a single researcher conducted the screening and data extraction. However, we attempted to limit the potential bias of this through conducting a 10% title check and then at the abstract screening stage all three reviewers discussed the excluded and ‘undecided’ papers.

## Conclusions

Findings from discrete choice experiments suggest that health-related claims have a substantial effect on dietary choices; however this effect varies according to the type of product. Further research is needed to see whether results may be replicated with similar claims and products. Furthermore, studies conducted in more natural settings suggest that health-related claims play a much smaller role in real-life dietary choices. Therefore, we highlight the need for more research into the effect of health-related claims on real-life dietary choices.

After taking these considerations and the findings of this review into account, it appears that health-related claims are likely to have a large effect on purchasing and consumption and, thus in turn, on public health. Given the prevalence of health-related claims and the concerns over ‘health halos’ it is important that health-related claims are regulated properly to ensure their validity so that only foods with a better nutritional composition may carry claims. Modelling exercises assessing the impact of using a nutrient profile are required.

Further work is also required to establish whether health-related claims lead to changes in dietary choices between products within a category (e.g. switching a cola drink for a fruit juice), or whether they increase total purchasing/consumption within a food category.

## Additional file

Additional file 1: Definitions and taxonomy used for the classification of health-related claims. Column headings used for data extraction. Search strategies used for MEDLINE, EMBASE, PsychINFO, CAB abstracts, Business Source Complete, and Web of Science/Science Citation Index & Social Science Citation Index. Data extracted for the risk of bias assessment. Completed PRISMA systematic review checklist. (ZIP 90 kb)

## Abbreviations

CI: Confidence intervals
Codex: Codex alimentarius commission
EU: European union
INFORMAS: International network for food and obesity/non-communicable disease research, monitoring and action support
Kcal: Kilocalories
OR: Odds ratios
UK: United Kingdom
WTP: Willing-to-pay
