# Supplementary material for: A systematic review, and meta-analyses, of the impact of health-related claims on dietary choices
Source: Int J Behav Nutr Phys Act. 2017 Jul 11;14:93. doi: 10.1186/s12966-017-0548-1 (PMC5505045; doi:10.1186/s12966-017-0548-1)
Supplement: Additional file 1: — Definitions and taxonomy used for the classification of health-related claims. Column headings used for data extraction. Search strategies used for MEDLINE, EMBASE, PsychINFO, CAB abstracts, Business Source Complete, and Web of Science/Science Citation Index & Social Science Citation Index. Data extracted for the risk of bias assessment. Completed PRISMA systematic review checklist. (ZIP 90 kb) [file 12966_2017_548_MOESM1_ESM.zip › IJBNPA SR Supplementary information 3 Search strategies.docx]

**A systematic review, and meta-analyses, of the impact of health-related claims on dietary choices**

Asha Kaur, Mike Rayner, Peter Scarborough. British Heart Foundation Centre on Population Approaches for Non-Communicable Disease Prevention, Nuffield Department of Population Health, University of Oxford.

**Supplementary information: Search strategies**

*Ovid Medline*

|  | **Searches** |
| --- | --- |
| 1 | (marketing/ or advertising as topic/) and (exp *Food/ or *beverages/ or carbonated beverages/ or energy drinks/) |
| 2 | food packaging/ or food labeling/ |
| 3 | ((food* or snack? or fat or fats or sugar? or salt? or sodium or sweet* or soda? or drink? or beverage?) adj5 (label* or pack*)).ti,ab. |
| 4 | ((nutrition* or nutrient?) adj5 (label* or pack*)).ti,ab. |
| 5 | ("back of pack*" or "front of pack*" or "on pack*").ti,ab. and (nutrition* or nutrient* or food* or snack* or fat or fats or sugar? or salt? or sodium or sweet* or soda* or drink* or beverage*).mp. |
| 6 | (*beverages/ or carbonated beverages/ or energy drinks/ or soda?.ti,ab. or carbonated drink?.ti,ab. or carbonated beverage?.ti,ab. or soft drink?.ti,ab.) and (label* or pack*).ti,ab. |
| 7 | (exp Food/ or food?.ti,ab. or snack?.ti,ab. or fat.ti,ab. or fats.ti,ab. or sugar?.ti,ab. or salt?.ti,ab. or sodium.ti,ab. or sweet*.ti,ab.) and (label* or pack*).ti,ab. |
| 8 | or/1-7 |
| 9 | (health-related claim? or logo? or symbol? or tick? or mark? or keyhole?).ti,ab. |
| 10 | 8 and 9 |
| 11 | (traffic adj3 light*).ti,ab. |
| 12 | ((color or colour) adj5 (code? or coding)).ti,ab. |
| 13 | 11 or 12 |
| 14 | 8 and 13 |
| 15 | 10 or 14 |

*Ovid PsychINFO*

|  | **Searches** |
| --- | --- |
| 1 | (exp marketing/ or advertising/ or retailing/) and (exp food/ or "beverages (nonalcoholic)"/) |
| 2 | exp Labeling/ or product design/ |
| 3 | exp food/ or food intake/ or diets/ or food preferences/ or nutrition/ or drinking behavior/ or eating behavior/ or health behavior/ or obesity/ or weight control/ |
| 4 | ((food* or snack? or fat or fats or sugar? or salt? or sodium or sweet* or soda? or drink? or beverage?) adj5 (label* or pack*)).ti,ab. |
| 5 | ((nutrition* or nutrient?) adj5 (label* or pack*)).ti,ab. |
| 6 | ("back of pack*" or "front of pack*" or "on pack*").ti,ab. and (nutrition* or nutrient* or food* or snack* or fat or fats or sugar? or salt? or sodium or sweet* or soda* or drink* or beverage*).mp. |
| 7 | (*beverages/ or carbonated beverages/ or energy drinks/ or soda?.ti,ab. or carbonated drink?.ti,ab. or carbonated beverage?.ti,ab. or soft drink?.ti,ab.) and (label* or pack*).ti,ab. |
| 8 | (exp Food/ or food?.ti,ab. or snack?.ti,ab. or fat.ti,ab. or fats.ti,ab. or sugar?.ti,ab. or salt?.ti,ab. or sodium.ti,ab. or sweet*.ti,ab.) and (label* or pack*).ti,ab. |
| 9 | 1 or 2 or 3 or 4 or 5 or 6 or 7 or 8 |
| 10 | (health-related claim? or logo? or symbol? or tick? or mark? or keyhole?).ti,ab. |
| 11 | 9 and 10 |
| 12 | (traffic adj3 light*).ti,ab. |
| 13 | ((color or colour) adj5 (code? or coding)).ti,ab. |
| 14 | 12 or 13 |
| 15 | 9 and 14 |
| 16 | 11 or 15 |

*OVID CAB 16.12.15*

|  | **Searches** |
| --- | --- |
| 1 | (exp marketing/ or exp food marketing/ or exp advertising/ or exp marketing techniques/ or exp food advertising/ or exp publicity/ or exp sales promotion/) and (exp food products/ or exp food/ or exp foods/ or exp beverages/ or exp lactic beverages/ or exp cocoa beverages/) |
| 2 | exp food packaging/ or exp labelling/ |
| 3 | ((food* or snack? or fat or fats or sugar? or sweet* or soda? or carbonated drink? or carbonated beverage? or soft drink?) adj5 (label* or pack*)).ti,ab. |
| 4 | ((nutrition* or nutrient?) adj5 (label* or pack*)).ti,ab. |
| 5 | ("back of pack*" or "front of pack*" or "on pack*").ti,ab. and (nutrition* or nutrient* or food* or snack* or fat or fats or sugar? or salt? or sodium or sweet* or soda* or drink* or beverage*).mp. |
| 6 | (*beverages/ or carbonated beverages/ or energy drinks/ or soda?.ti,ab. or carbonated drink?.ti,ab. or carbonated beverage?.ti,ab. or soft drink?.ti,ab.) and (label* or pack*).ti,ab. |
| 7 | (exp food/ or exp food products/ or exp food groups/ or exp meals/ or exp diet/) and (label* or pack*).ti,ab. |
| 8 | 1 or 2 or 3 or 4 or 5 or 6 or 7 |
| 9 | (health-related claim? or logo? or symbol? or tick? or mark? or keyhole?).ti,ab. |
| 10 | 8 and 9 |
| 11 | (traffic adj3 light*).ti,ab. |
| 12 | ((color or colour) adj5 (code? or coding)).ti,ab. |
| 13 | 11 or 12 |
| 14 | 8 and 13 |
| 15 | 10 or 14 |

*EMBASE 16.12.15*

|  | **Searches** |
| --- | --- |
| 1 | (exp advertizing/ or exp marketing/ or exp social marketing/) and exp food/ |
| 2 | exp food packaging/ |
| 3 | ((food* or snack? or fat or fats or sugar? or salt? or sodium or sweet* or soda? or drink? or beverage?) adj5 (label* or pack*)).ti,ab. |
| 4 | ((nutrition* or nutrient?) adj5 (label* or pack*)).ti,ab. |
| 5 | ("back of pack*" or "front of pack*" or "on pack*").ti,ab. and (nutrition* or nutrient* or food* or snack* or fat or fats or sugar? or salt? or sodium or sweet* or soda* or drink* or beverage*).mp. [mp=title, abstract, heading word, drug trade name, original title, device manufacturer, drug manufacturer, device trade name, keyword] |
| 6 | (*beverages/ or carbonated beverages/ or energy drinks/ or soda?.ti,ab. or carbonated drink?.ti,ab. or carbonated beverage?.ti,ab. or soft drink?.ti,ab.) and (label* or pack*).ti,ab. |
| 7 | (food/ or exp baby food/ or exp beverage/ or exp bran/ or exp cacao/ or exp canned food/ or exp cereal/ or exp chewing gum/ or exp condiment/ or exp cooked food/ or exp dairy product/ or exp dietary fiber/ or exp dough/ or exp edible oil/ or exp egg/ or exp fast food/ or exp fat/ or exp fat substitute/ or exp fermented product/ or exp food color/ or exp food composition/ or exp fruit/ or exp functional food/ or exp genetically modified food/ or exp gluten/ or exp health food/ or exp honey/ or exp margarine/ or exp meat/ or exp nectar/ or exp nut/ or exp organic food/ or exp pasta/ or exp raw food/ or exp roughage/ or exp sea food/ or exp sugar/ or exp vegetable/) and (label* or pack*).ti,ab. |
| 8 | 1 or 2 or 3 or 4 or 5 or 6 or 7 |
| 9 | (health-related claim? or logo? or symbol? or tick? or mark? or keyhole?).ti,ab. |
| 10 | 8 and 9 |
| 11 | (traffic adj3 light*).ti,ab. |
| 12 | ((color or colour) adj5 (code? or coding)).ti,ab. |
| 13 | 11 or 12 |
| 14 | 8 and 13 |
| 15 | 10 or 14 |

*Business source complete*

|  | **Searches** |
| --- | --- |
| 1 | marketing OR advertising |
| 2 | food OR beverages OR drink |
| 3 | S1 AND S2 |
| 4 | food packaging OR food labelling |
| 5 | ( ((food* OR snack? OR fat OR fats OR sugar? OR sweet* OR soda? OR carbonated drink? OR carbonated beverage? OR soft drink?) ) AND ( label* OR pack* ) |
| 6 | ( nutrition* OR nutrient? ) AND ( label* or pack* ) |
| 7 | ( "back of pack*" OR "front of pack*" OR "on pack*" ) AND ( nutrition* OR nutrient* OR food* OR snack* OR fat OR fats OR sugar? OR salt? OR sodium OR sweet* OR soda* OR drink* OR beverage* ) |
| 8 | ( beverages OR carbonated beverages OR energy drinks OR soda?.OR carbonated drink? OR carbonated beverage? OR soft drink? ) AND ( label* or pack* ) |
| 9 | ( food OR food products OR food groups OR meals OR diet) ) AND ( label* OR pack* ) |
| 10 | S3 OR S4 OR S5 OR S6 OR S7 OR S8 OR S9 |
| 11 | (health-related claim? OR logo? OR symbol? OR tick? OR mark? OR keyhole? |
| 12 | S10 AND S11 |
| 13 | traffic AND light |
| 14 | ( color OR colour ) AND ( code? or coding ) |
| 15 | S13 OR S14 |
| 16 | S10 AND S15 |
| 17 | S12 OR S16 |

*Web of Science*

|  | **Searches** |
| --- | --- |
| 1 | **TOPIC:** ((marketing/ or advertising) and (*Food/ or *beverages/ or carbonated beverages/ or energy drinks/))  Indexes=SCI-EXPANDED Timespan=All years |
| 2 | **TOPIC:** (food packaging or food labeling)  Indexes=SCI-EXPANDED Timespan=All years |
| 3 | **TOPIC:** (((food* or snack? or fat or fats or sugar? or salt? or sodium or sweet* or soda? or drink? or beverage?) NEAR/5 (label* or pack*)).)  Indexes=SCI-EXPANDED Timespan=All years |
| 4 | **TOPIC:** (((nutrition* or nutrient?) NEAR/5 (label* or pack*)))  Indexes=SCI-EXPANDED Timespan=All years |
| 5 | **TOPIC:** (("back of pack*" or "front of pack*" or "on pack*") and (nutrition* or nutrient* or food* or snack* or fat or fats or sugar? or salt? or sodium or sweet* or soda* or drink* or beverage*))  Indexes=SCI-EXPANDED Timespan=All years |
| 6 | **TOPIC:** ((*beverages/ or carbonated beverages/ or energy drinks/ or soda?.ti,ab. or carbonated drink?.ti,ab. or carbonated beverage?.ti,ab. or soft drink?) and (label* or pack*))  Indexes=SCI-EXPANDED Timespan=All years |
| 7 | **TOPIC:** ((Food/ or food? or snack? or fat or fats or sugar? or salt? or sodium or sweet*) and (label* or pack*))  Indexes=SCI-EXPANDED Timespan=All years |
| 8 | #7 OR #6 OR #5 OR #4 OR #3 OR #2 OR #1  Indexes=SCI-EXPANDED Timespan=All years |
| 9 | **TOPIC:** ((health-related claim? or logo? or symbol? or tick? or mark? or keyhole?))  Indexes=SCI-EXPANDED Timespan=All years |
| 10 | #9 AND #8  Indexes=SCI-EXPANDED Timespan=All years |
| 11 | **TOPIC:** (("traffic" NEAR/3 "light*"))  Indexes=SCI-EXPANDED Timespan=All years |
| 12 | **TOPIC:** (((color or colour) NEAR/5 (code? or coding)))  Indexes=SCI-EXPANDED Timespan=All years |
| 13 | #12 OR #11  Indexes=SCI-EXPANDED Timespan=All years |
| 14 | #13 AND #8  Indexes=SCI-EXPANDED Timespan=All years |
| 15 | #14 OR #10  Indexes=SCI-EXPANDED Timespan=All years |
