# Supplementary material for: A systematic review, and meta-analyses, of the impact of health-related claims on dietary choices
Source: Int J Behav Nutr Phys Act. 2017 Jul 11;14:93. doi: 10.1186/s12966-017-0548-1 (PMC5505045; doi:10.1186/s12966-017-0548-1)
Supplement: Additional file 1: — Definitions and taxonomy used for the classification of health-related claims. Column headings used for data extraction. Search strategies used for MEDLINE, EMBASE, PsychINFO, CAB abstracts, Business Source Complete, and Web of Science/Science Citation Index & Social Science Citation Index. Data extracted for the risk of bias assessment. Completed PRISMA systematic review checklist. (ZIP 90 kb) [file 12966_2017_548_MOESM1_ESM.zip › IJBNPA SR Supplementary information 4 Risk of Bias.docx]

**A systematic review, and meta-analyses, of the impact of health-related claims on dietary choices**

Asha Kaur, Mike Rayner, Peter Scarborough. British Heart Foundation Centre on Population Approaches for Non-Communicable Disease Prevention, Nuffield Department of Population Health, University of Oxford.

**Supplementary information: Risk of bias table**

| **First author (year)** | **How were participants recruited?** | **Were participants randomised to claim condition? Y/N** | **Were participants aware of claim allocation? Y/N** | **Were participants blind to study aims? Y/N** | **Were participants aware of study outcomes? Y/N** | **Were participants representative of the target population?** | **How was the study funded?** | **Any conflict of interests declared?** |
| --- | --- | --- | --- | --- | --- | --- | --- | --- |
| Ares (2008) | Randomly recruited in public areas (shopping areas, universities etc.). | NA - choice order was randomised. | NA. | Unclear. | Unclear. | Unclear - reported nutritional knowledge slightly lower than reported in some European studies. | Sensory Science Scholarship Fund. GlaxoSmithKline Consumer healthcare for the Rose Marie Pangborn Sensory Science Scholarship. | No conflict of interest statement. |
| Ares (2009) | Randomly recruited in public areas (shopping areas, universities etc.). | NA - choice set was not randomised to reduce contamination. | Unclear. | Unclear. | Unclear. | Unclear. | Sensory Science Scholarship Fund. GlaxoSmithKline Consumer healthcare for the Rose Marie Pangborn Sensory Science Scholarship. | No conflict of interest statement. |
| Ares (2010) | Randomly recruited in public areas (shopping areas, universities etc.). | NA - all participants completed the same 18 choice sets. | NA. | Unclear. | Unclear. | Yes. | Sensory Science Scholarship Fund. GlaxoSmithKline Consumer healthcare for the Rose Marie Pangborn Sensory Science Scholarship. | No conflict of interest statement. |
| Aschemann-Witzel (2010) | Unclear. | NA. | NA. | Unclear. | Unclear. | Mixed - age and gender quota sampling but persons with a higher education level were over-represented. | The empirical research was funded by the German Research Foundation (DFG). | No conflict of interest statement. |
| Aschemann-Witzel (2013) | Randomly recruited in public areas (shopping areas, universities etc.). | Yes - claim set rotated equally between participants. | NA. | Unclear - asked to choose one brand. | Yes - told they will be asked to choose (or not) a product. | Yes - age and gender quota sampling | German Federal Ministry of Food, Agriculture and Consumer Protection in the frame of the federal funding scheme for organic agriculture (BÖL, FKZ: 06OE120). | No conflict of interest statement. |
| Barreiro-Hurle (2010) | Randomly recruited in public areas (shopping areas, universities etc.). | NA - 80 choice sets randomly split into 20 blocks, each participant one block of four choice sets. | NA. | Unclear. | Unclear. | Yes - stratified random sampling by town and age. Town socio-demographics representative of the Spanish Census of Population (INE, 2004). | DISOPTIPOL project funded by INIA-MICINN and EU FEDER through research grant RTA2005-0020. JBH undertook this research while contracted under the INIA-CCAA cooperative research system post-doctoral incorporation scheme, partly funded by EU-ESF. | No conflict of interest statement. |
| Belei (2012) | Unclear. | Yes. | Unclear. | Unclear. | Unclear. | Unclear. | European Union (Marie Curie Individual Fellowship Grant 254931), Netherlands Organization for Scientific Research (Veni Individual Grant 451- 10-009), and Marketing Science Institute (Grant 4-1743). | No conflict of interest statement. |
| Carbonneau (2015) | Various advertisements (e.g. flyers, forum postings, email, etc.). | Yes. | Unclear. | Yes - told that this study aimed to rate the appreciation of a new 7 day menu over a 10 day period. | Mixed - asked to return uneaten food but not told it would be weighed. | Unclear. | Canadian Institutes of Health Research (CIHR) (grant no. MOP-110951). | Authors state that there are no conflicts of interest. |
| Casini (2014) | Recruited in-person. | NA - 16 choice situations divided into two blocks of eight sets. | NA. | Unclear. | Unclear. | No. | Unclear. | No conflict of interest statement. |
| Coleman (2014) | Research panel | NA - same choice set. | NA. | Unclear. | Unclear. | Unclear. | Unclear. | Authors state that there are no conflicts of interest. |
| Contini (2015) | Unclear. | NA - 64 choice pairs divided into 4 blocks of 16 sets. | NA. | Unclear. | Unclear. | Yes - sample representative for age, gender, and education (and consumers of olive oil). | Unclear. | No conflict of interest statement. |
| De Marchi (2016) | Research panel | NA. | NA | No - cheap talk script. | Yes - cheap talk script. | Unclear. | Tyson Chair Endowment at the University of Arkansas, by the National Research Foundation of Korea (Grant NRF-2014S1A3A2044459) and by the Research Council of Norway (Grant-233800) | No conflict of interest statement |
| De-Magistris (2016) | External agency | NA | NA | No – informed consent. | Yes – informed consent. | Yes - random stratified sampling according to gender, age, and BMI. | European Commission FP7-MC-CIG-332 769, Fighting against obesity in Europe: the role of health-related claim in food products (OBESCLAIM). | Authors state that there are no conflicts of interest. |
| Fernández-Polanco (2013) | Randomly recruited in public areas (shopping areas, universities etc.). | NA - choice order was randomised. | NA. | Mixed - told that investigating consumer preferences for fish. | Mixed - told that investigating consumer preferences for fish. | Yes. | Unclear – “The authors wish to acknowledge the following persons and institutions: the board and members of the ‘‘Mercado de la Esperanza’’ (Santander, Spain) retailers’ association for their inestimable cooperation and support in the field work of this and other researches.” | No conflict of interest statement. |
| Gracia (2009) | Unclear. | NA - choice order was randomised. | NA. | Unclear. | Unclear. | Yes - stratified random sampling design based on town and age. | Unclear. | No conflict of interest statement. |
| Kiesel (2013) | Unclear - authors were not given information on how stores were selected. | NA. | NA. | NA. | NA. | Authors were not given information on how the five stores were selected. | Giannini Foundation. | No conflict of interest statement. |
| Koenigstorfer (2013) | Recruited in university hallway. | Unclear. | Unclear. | Yes. | No. | Unclear - University students. | Postdoc- Programme of the German Academic Exchange Service (DAAD). | No conflict of interest statement. |
| Kozup (2003) | Research panel | Unclear. | Unclear. | Unclear. | Unclear. | Unclear. | Unclear if University funded. | No conflict of interest statement. |
| Krystallis (2012) | Research panel | NA - choice order was randomised. | NA. | Unclear. | Unclear. | Unclear. | Unclear. | No conflict of interest statement. |
| Lin (2015) | Convenience sampling. | Yes. | Unclear. | Unclear. | Unclear. | Unclear. | Unclear. | No conflict of interest statement. |
| Loose (2013) | Research panel | NA - choice order was randomised. | NA. | Unclear - asked about oyster preference. | Unclear - asked about oyster preference. | Maybe - females and older participants slightly over-represented compared to other seafood research (Olsen, 2003). | Australia’s Fisheries Research and Development Cooperation (FRDC). | No conflict of interest statement. |
| Maubach (2014) | Research panel | NA - choice order was randomised. | NA. | No - informed consent. | Unclear. | Unclear. | Department of Marketing at the University of Otago. | Authors state that there are no conflicts of interest. |
| McLean (2012) | Research panel | NA - choice order was randomised. | NA. | Unclear. | Unclear. | Random selection from national research panel 50% with/without hypertension. | Health Research Council of New Zealand through the University of Otago. | Authors state that there are no conflicts of interest. |
| Mohebalian (2012) | Research panel | NA. | NA. | Unclear. | Unclear. | Yes - participant data fit well with census data. | Unclear. | No conflict of interest statement. |
| Mohebalian (2013) | Research panel | NA. | NA. | Unclear. | Unclear. | Yes - participant data fit well with census data. | Partial funding: Sustainable Agriculture Research and Education (SARE) grant, LNC10-324. | No conflict of interest statement. |
| Moon (2011) | Research panel | Yes. | Unclear. | Unclear. | Unclear. | Yes. | Illinois Missouri Biotechnology Alliances (IMBA). | No conflict of interest statement. |
| Orquin (2015) | Research panel | Studies 1-3: Yes. | Unclear. | Yes - All experiments had cover stories to minimize demand characteristics. | Unclear. | Unclear. | Partial funding: Danish Council for Strategy Research (grant 2101-09-044- "Bridging the gap between health motivation and food choice behaviour: A cognitive approach" (HEALTHCOG)). | No conflict of interest statement. |
| Roberto (2012) | Various advertisements (e.g. flyers, forum postings, email, etc.). | Yes - stratified by gender. | Unclear. | Unclear - told they would be asked to taste a cereal and provide feedback. | No- asked filler questions about other aspects of the cereal. 27 participants excluded as correctly identified study was testing influence of nutrition information. | Unclear. | Rudd Foundation and Robert Wood Johnson Foundation. | Authors state that there are no conflicts of interest. |
| Steenhuis (2010) | Various advertisements (e.g. flyers, forum postings, email, etc.). | NA - choice order was randomised. | Unclear. | Yes - experiment was presented as a product evaluation study. | No - true purpose of the experiment was not revealed to the respondents until the entire experiment was finished. | Unclear. | Unclear. | No conflict of interest statement. |
| Van Wezemael (2014) | Research panel. | Yes. | Unclear. | Unclear. | Yes - participants told about the attributes that were included in the study. | Yes - representative for each of the national populations in terms of gender and age. | EU FP6 Integrated Project ProSafeBeef, Contract No. FOOD-CT-2006-36241 and the Agricultural University of Athens. | No conflict of interest statement. |
| Wansink (2006) | Recruited at a University. | Study 1: No. Study 3: Unclear. | Unclear. | Study 1: Mixed - participants asked if they wanted to be involved in a series of demonstrations and short surveys about how consumers make choices and decisions. Study 3: Yes - participants asked to evaluate a pilot episode for a television show. | Study 1: Unclear. Study 3: No. | Study 1: Unclear. Study 3: Unclear. | Funding source not given but authors declare that "No industry or government agency funds sponsored this project." | No conflict of interest statement. |
