# Supplementary material for: A systematic review, and meta-analyses, of the impact of health-related claims on dietary choices
Source: Int J Behav Nutr Phys Act. 2017 Jul 11;14:93. doi: 10.1186/s12966-017-0548-1 (PMC5505045; doi:10.1186/s12966-017-0548-1)
Supplement: Additional file 1: — Definitions and taxonomy used for the classification of health-related claims. Column headings used for data extraction. Search strategies used for MEDLINE, EMBASE, PsychINFO, CAB abstracts, Business Source Complete, and Web of Science/Science Citation Index & Social Science Citation Index. Data extracted for the risk of bias assessment. Completed PRISMA systematic review checklist. (ZIP 90 kb) [file 12966_2017_548_MOESM1_ESM.zip › IJBNPA SR Supplementary information 1 Claim definitions.docx]

**A systematic review, and meta-analyses, of the impact of health-related claims on dietary choices**

Asha Kaur, Mike Rayner, Peter Scarborough. British Heart Foundation Centre on Population Approaches for Non-Communicable Disease Prevention, Nuffield Department of Population Health, University of Oxford.

**Supplementary material: Health-related claim definitions**

| **Claim type** | **Definition** | **Example** |
| --- | --- | --- |
| Health claim | ‘any claim which states, suggests or implies that a relationship exists between a food category, a food or one of its constituents and health’^^[[1]](#footnote-1)^^. | |
| General health claim | claims concerning the general beneficial effects of the consumption of foods or their constituents on health. | ‘healthy goodness’ |
| Nutrient and other function claims | claims that refer to the function of a nutrient or other substance in the growth/development/function of the body. ^3^ | ‘contains calcium…good for healthy bones’ |
| Reduction of disease risk claims | claims that refer to a food (or one of its constituents and an associated reduced risk in developing a disease or health-related condition.^3^ | ‘contains calcium...reduces risk of osteoporosis’ |
|  |  |  |
| Nutrition claim | ‘any claim that states, suggests or implies that a food has particular beneficial nutritional properties due to the energy, nutrients or other substances it contains, contains in reduced or increased proportions or does not contain’^2^ | |
| Nutrient content claims | claims that refer to the amount of a nutrient in a product | ‘low in fat’ |
| Nutrient comparative claims | claims that compare the nutrient levels between two or more products, or the same product that has been reformulated | ‘reduced salt’  ‘lower in salt than x’ |
| Health-related ingredient claims | health-related claims that refer to substances other than nutrients or energy | ‘contains wholegrain’ |
|  |  |  |
| Symbolic claim – these were excluded from the systematic review if there was not publicly available criteria governing its use, or an explanation of its criteria was not provided. | a pictorial, or combined pictorial and text, health-related claim for which there are criteria underpinning its use. | Symbolic general health claim: Dutch ‘Choices’ logo^^[[2]](#footnote-2)^^  Symbolic nutrient and other function claims: ‘Toothfriendly’ logo^^[[3]](#footnote-3)^^ |

1. European Union (2006). Regulation No 1924/2006 on nutrition and health claims made on foods. *Official Journal of the European Union* L404/9. [↑](#footnote-ref-1)
2. http://www.choicesprogramme.org/ [↑](#footnote-ref-2)
3. http://www.toothfriendly.org/en/certification [↑](#footnote-ref-3)
