# Supplementary material for: A systematic review, and meta-analyses, of the impact of health-related claims on dietary choices
Source: Int J Behav Nutr Phys Act. 2017 Jul 11;14:93. doi: 10.1186/s12966-017-0548-1 (PMC5505045; doi:10.1186/s12966-017-0548-1)
Supplement: Additional file 1: — Definitions and taxonomy used for the classification of health-related claims. Column headings used for data extraction. Search strategies used for MEDLINE, EMBASE, PsychINFO, CAB abstracts, Business Source Complete, and Web of Science/Science Citation Index & Social Science Citation Index. Data extracted for the risk of bias assessment. Completed PRISMA systematic review checklist. (ZIP 90 kb) [file 12966_2017_548_MOESM1_ESM.zip › IJBNPA SR Supplementary information 2 Column headings.docx]

**A systematic review, and meta-analyses, of the impact of health-related claims on dietary choices**

Asha Kaur, Mike Rayner, Peter Scarborough. British Heart Foundation Centre on Population Approaches for Non-Communicable Disease Prevention, Nuffield Department of Population Health, University of Oxford**.**

**Supplementary information: Column headings used for data extraction**

| Notes: |
| --- |
| # ID number |
| First author (year) |
| Title |
| IN/OUT |
| Reason for exclusion |
| Notes |
| Brief description of study (or abstract) |
| Study aim |
| Country |
| Study type |
| Study design |
| Setting |
| Study powered |
| Population |
| How were participants recruited? |
| Were participants randomised to claim condition? Y/N |
| Were participants aware of claim allocation? Y/N |
| Were participants blind to study aims? Y/N |
| Were participants aware of study outcomes? Y/N |
| Were participants representative of the target population? |
| How was the study funded? |
| Any conflict of interests declared? |
| Risk of bias notes |
| Method |
| Analysis |
| Health/Nutrition/Logo Claim |
| Claim sub-type (nutrient/target - health relationship) |
| Claim details |
| Eatwell |
| sub-eatwell |
| Comparator |
| Outcome measure |
| Results |
| No choice option? |
| adjustments/interactions |
| How much completed outcome data was obtained? |
| Other (non-SR) results |
| Overall, do health claims increase consumption/purchase |
| Overall, do nutrition claims increase consumption/purchase? |
| Does it support the hypothesis that claims increase purchases/consumption? Y/N |
